# The effect of a collaborative approach to implementing music-based interventions for patients with different neurological disorders

**DOI:** 10.3389/fpsyt.2026.1736793

**Published:** 2026-02-27

**Authors:** Li-Jie Du, Yun-Ze Li, Xing-Yuan Du

**Affiliations:** 1 Department of Sociology, College of Philosophy and Law and Politics, Shanghai Normal University, Shanghai, China; 2 School of Mathematics and Information Science, Nanjing Normal University of Special Education, Nanjing, China; 3 School of Journalism and Communication, University of Chinese Academy of Social Sciences, Beijing, China

**Keywords:** depression, effect, music intervention, music-based intervention, neurological disorder, patients

## 1 Introduction

Throughout history, music has served as a healing practice to treat ailments and nourish the mind. It has been an integral part of human life. Music healing, which describes the holistic effects of music, can be classified into various medical approaches, including music medicine, music therapy, and other music-based interventions (1). In the early 19th century, its highly expressive nature led psychiatrists to believe that listening to Romantic music could alter patients’ mental states. Horden argued that music therapy arose as a result of institutional psychiatry and Romantic music (2). However, Romantic music is not well-defined, and what qualifies as Romantic music is a controversial issue, with no consensus.

Music-based intervention is a broader concept encompassing music medicine, music therapy, and other music-related interventions, where music therapy specifically refers to an intervention developed by a certified music therapist. According to the American Music Therapy Association (AMTA), music therapy is the “clinical and evidence-based use of music to accomplish individualized goals within a therapeutic relationship by a certified professional who has completed an approved music intervention program” (3). Drawing on previous literature, this article highlights the effects of applying music-based interventions for patients with various neurological disorders. The cited literature spans multiple contexts and formats, where terms such as “music intervention” and “music medicine” are often used interchangeably. To ensure consistency, this article uniformly employs “music intervention” instead of retaining the terminology specific to each original context.

This article advocates for a collaborative approach to implementing music-based interventions and acknowledges that different cultures and approaches do exist. It culminates in a discussion of two critical observations. First, “vertical collaboration” (cross-cultural or cross-group) in music interventions ensures cultural adaptability, with the cultural characteristics deeply rooted in ethnic and geographical determinants. Second, “horizontal collaboration” lies in knowledge integration and tackles complexity through a multidisciplinary perspective. The maximal efficacy of music interventions is contingent upon their integration with other therapeutic paradigms.

## 2 Music-based interventions effectively address various neurological disorders while enhancing mental health and well-being across diverse populations

Music intervention has been proven to be significantly effective for individuals with sleep disorders (4, 5), depression (6), stroke (7), and Alzheimer’s disease (7). It can also improve the psychological state of cancer patients and alleviate pain, among other benefits (8). The application of music to enhance the physical and mental well-being of mothers and infants during and after pregnancy constitutes an important component of the music intervention discipline (9–11). The research findings presented below provide robust evidence for the positive effects of music-based interventions in addressing neuropsychiatric disorders and broader mental health or well-being outcomes for populations without disorders.

### 2.1 Music-based interventions for insomnia disorder

Insomnia Disorder (ID) is a common sleep disorder in modern society, and listening to music is a widely used sleep aid. A music-based intervention in this case is safe and easy to administer, and the primary objective of randomized and quasi-randomized controlled trials is to compare the effects of listening to music with no treatment or treatment-as-usual (TAU) on improving sleep in individuals with insomnia. The findings provide evidence that music may be effective in improving subjective sleep quality in adults with insomnia (12).

Insomnia is also a common sleep disorder among adults with depression. It reduces quality of life with significant negative effects on daytime cognitive function, and it is associated with impairments in physical and mental health. Based on comparisons of a music intervention plus TAU vs. TAU alone, the findings indicate that the combination is beneficial for patients with depression in the short term compared to TAU alone, while also improving anxiety and functioning (4).

Previous trials have suggested that listening to music may be helpful in the treatment of sleep disturbances in healthy populations, including students and older adults. In addition, small-scale studies with clinical populations of traumatized refugees, adults with chronic insomnia, and adults with depressive insomnia add to the evidence base (13). Music that aids sleep is not necessarily the “sedative music”, as defined in academic literature; however, certain features of sedative music are indeed linked to sleep-promoting effects. A few studies have sought to identify the specific musical characteristics reported to be effective or ineffective in aiding sleep. Pinpointing these traits is critical for selecting musical pieces for music-based intervention research (14).

Sleep is a complex rhythmic state that may be affected by the aging process. A few studies have focused on the effects of music as a non-pharmacological method of improving the quality of sleep in older adults. One study investigated the effects of soft music on sleep quality in older community-dwelling men and women in Taiwan. Music was found to significantly improve sleep quality in the experimental group. Sleep was also found to improve every week, indicating a cumulative dose effect. These findings provide evidence for the use of soothing music as an empirically-based intervention for sleep in older adults (5). These studies suggest that more research is required to strengthen the scientific knowledge about the effects of sleep quality in adults with sleep disturbances (11).

### 2.2 Music for stroke survivors, cancer patients, and those with Alzheimer’s disease

Research findings have advocated for the use of music in stroke rehabilitation, cancer care, and pain management. When integrated into routine stroke rehabilitation, music intervention approaches have received excellent feedback from both patients and caregivers. All caregivers expressed belief that this therapy could improve stroke rehabilitation practices and yield tangible benefits for the patients (7).

Research has also indicated that a music intervention can enhance cognition, mood, and behavior in individuals with AD. Non-pharmacological therapy has gained popularity in the treatment of Alzheimer’s disease (AD) due to its apparent therapeutic effectiveness and the shortcomings of biological drugs (15). Research on music interventions for dementia in the United States began in the mid-1980s, and music intervention has been applied as a broader therapeutic approach for dementia since the 1990s (16).

Music-based interventions may also reduce anxiety, depression, and pain catastrophizing. The data support the use of music-based interventions to reduce postoperative opioid requirements. Patients in a music-based intervention group consumed fewer opioids than those in the control group (17).

One study has highlighted the role of music in cancer care, indicating that patient characteristics—such as their outlook on life and willingness to explore emotions tied to their cancer experience—can influence treatment outcomes. Quantitative data further reveal that both music therapy (MT) and music medicine (MM) interventions are equally effective in improving psychological outcomes and reducing pain in cancer patients (8). A meta-analysis has demonstrated that listening to music significantly alleviates preoperative anxiety and postoperative pain in patients undergoing gynecological surgery, compared with the control group. Consequently, perioperative implementation by operating room nurses is recommended (18).

### 2.3 Music-based interventions can help alleviate the stress of pregnancy in women

The transition to motherhood can be a stressful journey, but women’s psychological and emotional needs often take a backseat to their physical well-being. Pregnancy profoundly impacts a woman’s physical and mental health, and caring for a newborn after childbirth presents unique challenges. Stress and anxiety are common during both pregnancy and the postpartum period, with detrimental effects on both mothers and infants. Existing research in this area has provided evidence supporting the use of music-based interventions to promote maternal mental health among women with healthy term infants during the postpartum phase (9). This type of therapy aims to reduce stress and anxiety levels, provide emotional support, and enhance maternal-infant bonding for women during antepartum and postpartum hospitalization at a large urban medical center. An evaluation study has shown that a music intervention program was implementable and well-received, with high satisfaction reported by the participants, positive effects on their relaxation and sense of connection with their infants, and strong endorsement from involved healthcare providers and staff (19). Gestational hypertension is a common complication during pregnancy. A systematic review and meta-analysis have suggested that adjunctive music intervention programs provide clinically relevant benefits for controlling systolic and diastolic blood pressure and improving psychological outcomes, including anxiety, depression, and sleep quality, in patients with hypertensive disorders of pregnancy(HDP) when compared with standard care alone (10).

Prenatal sleep disturbance has been associated with undesirable birthing outcomes. The effectiveness of listening to music at home in improving sleep quality was determined in a study about Taiwanese pregnant women with poor sleep quality. This study supported the theory that 2-week music listening interventions may reduce stress, anxiety, and yield better sleep quality in sleep-disturbed pregnant women (11).

## 3 Discussion

The broad applicability of music-based interventions across diseases and populations arises from their universal regulatory role via a “biology-psychology-society” multidimensional mechanism. When applied to heterogeneous groups, its standardized implementation typically follows three sequential stages: Needs Assessment (physical/psychological indicators) → Program Customization (music type/intervention form) → Effect Feedback (quantitative + qualitative data). While this framework ensures procedural standardization, enhancing multiparty collaboration remains critical to improving intervention outcomes. Music-based interventions lie in respecting the “root” of cultural diversity and pursuing the “integration” of therapeutic approaches, viewed from a collaborative perspective. This usually requires two forms of collaboration: “vertical collaboration” (cross-cultural or cross-group) to ensure its cultural adaptability, and “horizontal collaboration” (cross-disciplinary or cross-methodological) to achieve precise, systematic treatment.

### 3.1 The core of vertical collaboration is cultural rootedness, requiring programs to be deeply bound to the historical traditions, cultural symbols, and aesthetic preferences of specific groups

The cultural characteristics embedded in music-based interventions are deeply rooted in ethnic and geographical determinants. Any music intervention possesses distinct regional cultural traits, as manifested by how musical traditions from different areas influence therapeutic approaches and outcomes. North American music, characterized by its diversity and fusion with modern genres, widely employs methods such as improvisation and music composition in therapy, reflecting the practice of the Nordoff-Robbins music intervention approach. Middle-Eastern music is frequently applied in meditation, religious rituals, and emotional regulation, reflecting local religious and cultural customs. Ancient Persian Medicine(PM) always takes into account the patient’s cultural background and region, and adjusts prescriptions accordingly. This practice even affects music intervention programs, which change based on individual patient differences. This can be described as a form of ethnomusical intervention (1), which originates from music intervention, and is performed by a certified professional who considers the ethnic and cultural properties of music in the healing process (20). The cross-cultural implementation of music interventions requires the establishment of a professional collaborative team, whose core members should include music therapists, cultural anthropologists, and local medical staff.

For instance, Persian medical experts can be invited to verify the compatibility between music intervention programs and local traditional medical theories. In the music selection process, a dynamic screening mechanism should be established to accurately match the type of disease and intervention goals. Jazz standards can be used to promote social interaction among depressed elderly patients in the United States, while Oud improvisation can be adopted to distract patients with chronic pain in the Middle East from their pain through the linear flow of melody. To ensure the cultural fidelity of the intervention, it is recommended to introduce the “Cultural Congruence Scale”, in which local experts evaluate the compatibility of musical elements with the target culture—for example, when using African-American spirituals, their cultural appropriateness needs to be verified through community religious context assessment tools, ultimately achieving the deep integration of intervention programs with community culture. In special education, a music intervention can also be employed to assist children with autism. Cross-cultural comparative studies have indicated that music intervention methods and outcomes for children vary across cultural contexts. Within folklore and child psychology traditions, both descriptive and empirical research confirms that lullabies and play songs serve not only as caregiving tools but also constitute vital components of regional knowledge systems (21).

Kerry Byers (2016) claimed that music therapists returning from studying in the U.S. and U.K. introduced music intervention to the majority of countries, but each country’s development of music intervention deserves independent inquiry (22). That study examined the effectiveness of music intervention programs on depressive symptoms among care home residents with dementia in Australia, Germany, the Netherlands, Norway, Türkiye, and the U.K. Interpretation of the findings indicates that effects varied between countries. Notably, Country was the strongest predictor of differences in effects, underlining the importance of cultural and systemic differences (23). Globally, intervention guidelines and healthcare policies must be carefully tailored to the specific cultural and systemic contexts of care home populations and varying levels of care in different countries and regions.

### 3.2 The essence of horizontal collaboration lies in knowledge integration, which tackles complexity through a multidisciplinary perspective

Since their inception, music-based interventions have merged with other methods, serving together as a resource to promote human mental health and well-being. Since the 1990s, the “Creative music intervention” proposed by Paul Nordoff and Clive Robbins, and Tom Kitwood’s “person-centered dementia care” approach have shared humanistic therapeutic principles, forming a convergence of theories. This alignment has promoted the adoption of music intervention as a broader therapeutic approach for dementia in the United States (16). The latest findings from Europe, implemented across Italy, Portugal, and Romania, have shown that SOUND, a person-centered intervention delivered to 41 older adults with mild-to-moderate dementia in elder care facilities, resulted in significant improvements in participants’ well-being, cognition, and executive functions over time, with these benefits remaining stable in cross-national follow-up assessments (24).

Although music-based interventions have demonstrated significant efficacy in treating patients with single-disease conditions with substantial supporting evidence, they exhibit certain limitations when applied to patients with complex conditions, where standalone interventions yield less pronounced effects. For instance, in the management of chronic pain in individuals with dementia, various types of music intervention have been recommended and utilized as an alternative to analgesic drugs (17); however, relevant evidence remains notably insufficient. The treatment of chronic pain in patients with dementia is challenging because they have a reduced ability to report pain and are particularly vulnerable to the side effects of analgesics. A cluster randomized controlled trial (RCT) has aimed to demonstrate that an 8-week music-based care intervention (MBC) can effectively reduce pain intensity among nursing home residents with dementia and chronic pain. Unfortunately, this reliable randomized controlled study did not find that music care had an effect on the pain intensity of nursing home residents with dementia and chronic pain (25).

To improve upon the above research, we should explore potential improvement factors by examining intervention dosage and implementation fidelity. This is because an insufficient sample size or sub-threshold intervention intensity may prevent the full manifestation of effects. Simultaneously, collaboration across multiple domains and disciplines should be strengthened. First, it is recommended to optimize intervention protocols through multi-stakeholder collaboration involving physicians, patients, family members, and pharmacists; this can be achieved by implementing combined non-pharmacological therapies. Second, incorporating professional recommendations from collaborative teams, including neurologists and rehabilitation therapists, and introducing objective indicators such as cortisol levels and sleep quality to enhance the sensitivity of outcome assessments. The maximal efficacy is contingent upon its integration with other therapeutic paradigms.

Music-based interventions work best when combined with other approaches. Given the existing evidence demonstrating that music intervention can be effectively integrated with social work, its significance in clinical treatment should not be overlooked. Therefore, it is essential to enhance attention to the elderly psychiatric inpatients, improve the mechanism integrating music intervention with social work, and promote multidisciplinary collaboration (26). Previous studies have suggested that mindfulness-based music listening (MBML) can effectively improve conflict control and attentional processing in healthy adults. Therefore, listening to music while maintaining mindfulness can enhance positive emotional experiences and improve cognitive abilities, thereby reducing excessive anxiety levels among young adults with insomnia (27). Music-based intervention programs advocate for a collaborative and holistic model, while acknowledging the necessity of different approaches. It may be necessary to explore differentiated pathways, tailored to individual needs and expectations that are most meaningful to the patients (28).

Music-based interventions functionally integrate diverse therapeutic paradigms—such as person-centered care, social work, and mindfulness-based approaches through a three-dimensional synergistic framework. The physiological-psychological-social triple-axis framework clarifies the role division of each paradigm: (1) Physiologically: Music intervention modulates heart rate variability (HRV) via auditory-motor cortex synchronization, while mindfulness stabilizes the autonomic nervous system. Together, they synergistically optimize the neuroendocrine-immune network. (2) Psychologically: Person-centered care offers a safe space for expression; music therapy enables emotional release through melody/lyrics; and social work addresses real-world stressors. (3) Socially: Social work connects community resources (e.g., music group activities); mindfulness enhances social adaptability; music therapy mediates group interaction to strengthen social support networks. Finally, integrating multidimensional assessments with longitudinal tracking enables systematic confounding effect control, providing a verifiable theoretical framework and an empirical basis for the integration of interdisciplinary music interventions.

## 4 Conclusion

Music-based interventions have demonstrated efficacy in managing a broad spectrum of neurological and psychiatric conditions. As a recognized therapeutic modality, music intervention has been utilized in clinical settings for decades. Beyond their effectiveness in addressing various neurological and psychiatric disorders, music-based interventions also exhibit distinct characteristics shaped by cultural contexts. Research prioritizing the psychosocial benefits of music over purely musical outcomes is increasingly prevalent (29). Future research efforts should focus on standardizing outcome measures, intervention delivery settings, implementation methods, and target populations to improve the practical efficacy of music interventions. These endeavors aim to contextualize findings within clinical frameworks, guide evidence-based practices, and further establish music intervention as a validated therapeutic approach (4). Additionally, there is a critical need for large-scale, cross-national studies that compare the ethnographic dimensions of music, in addition to integrated research that combines biological, molecular testing with socio-psychological assessments in music-based interventions for patients with different neurological disorders.
